# Medullary ischemia after endovascular procedure of infrarenal aorta in a patient with dual anticoagulant and antiplatelet therapy: a case report

**DOI:** 10.1186/s13256-019-2168-7

**Published:** 2019-08-05

**Authors:** Erika D. Pérez-Riveros, Cesar A. Cardona-Montes, Carlos A. Zapata-Álvarez, Wendy L. Sotelo-Hernández, Alirio R. Bastidas-Goyes

**Affiliations:** Clínica Universidad de la Sábana, Chía, Colombia

**Keywords:** Spinal cord ischemia, Blood vessel prosthesis, Platelet aggregation inhibitors, Anticoagulants

## Abstract

**Background:**

Medullary ischemia secondary to surgical procedures of the infrarenal aorta is an infrequent and mostly devastating complication of this procedure, and its nonspecific clinical presentation makes it difficult to promptly diagnose. Prevention measures for this complication are not yet clear; therefore, the need for anticoagulant and/or antiplatelet therapy is discussed.

**Case presentation:**

This paper reports a case of a 69-year-old Hispanic man presenting with sudden pain and signs of ischemia on his left lower extremity 8 weeks after endovascular repair with endoprosthesis of an infrarenal aorta and left iliac aneurysm. The patient was admitted to the emergency room, where an extensive arterial thrombosis compromising the right iliac and femoral arteries was diagnosed. Dual anticoagulation and antiplatelet therapies were initiated, and therapeutic ranges were achieved. Nonetheless, the patient presented medullary ischemia by microembolization diagnosed by contrast-enhanced magnetic resonance imaging, with unsatisfactory evolution of an intracranial hemorrhagic event without documented excessive anticoagulation. The patient developed permanent pure motor deficit of his lower extremities, absence of sphincter control, and mild cognitive impairment.

**Conclusions:**

This is a complex and extremely rare case. It is important to continue with clinical investigations that give more clarity about the onset of anticoagulation, antiplatelet therapy, and management of dual schemes to decrease the risk of complications in this type of surgical procedure.

## Introduction

Medullary ischemia secondary to surgical procedures of the infrarenal aorta has been reported in the literature as an infrequent complication; however, when present, it can be catastrophic [1]. The diagnosis of this complication is mostly clinical and achieved with the aid of imaging [2]. In several reviews in the literature, it is considered that this event may be preventable with adequate postoperative care; however, the need for anticoagulant and/or antiplatelet therapy is discussed in several clinical studies [3]. Despite not having a clear indication, it is thought that in certain patients it is necessary to consider prescribing at least one antiplatelet medication in order to reduce prothrombotic risks [4]. This paper reports the case of a 69-year-old man who underwent an endovascular repair of an infrarenal aorta and left iliac aneurysm, and even after receiving anticoagulant and antiplatelet therapy, both within therapeutic ranges, he had multiple thromboembolic, ischemic, and hemorrhagic complications 8 weeks after his surgery, with permanent sequelae.

## Case presentation

**Fig. 1:**
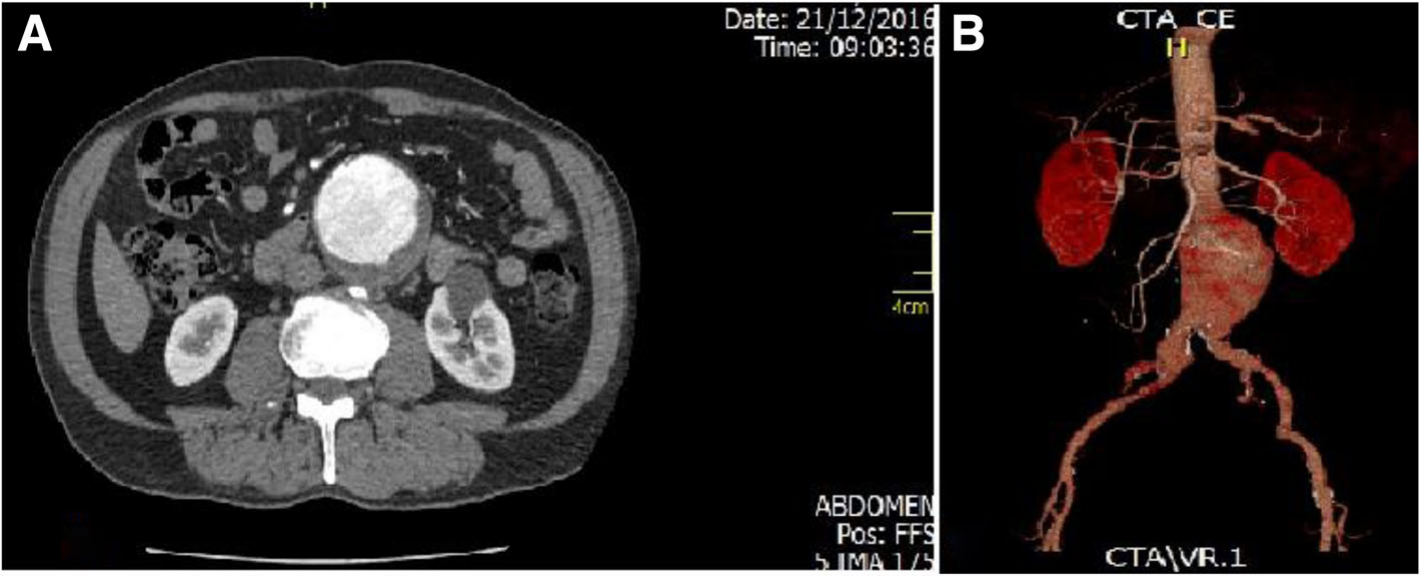
**a** Abdominal computed tomographic (CT) scan in transverse section prior to endovascular surgery. An infrarenal aortic aneurysm is observed. **b** CT angiography showing infrarenal aortic aneurysm

**Fig. 2:**
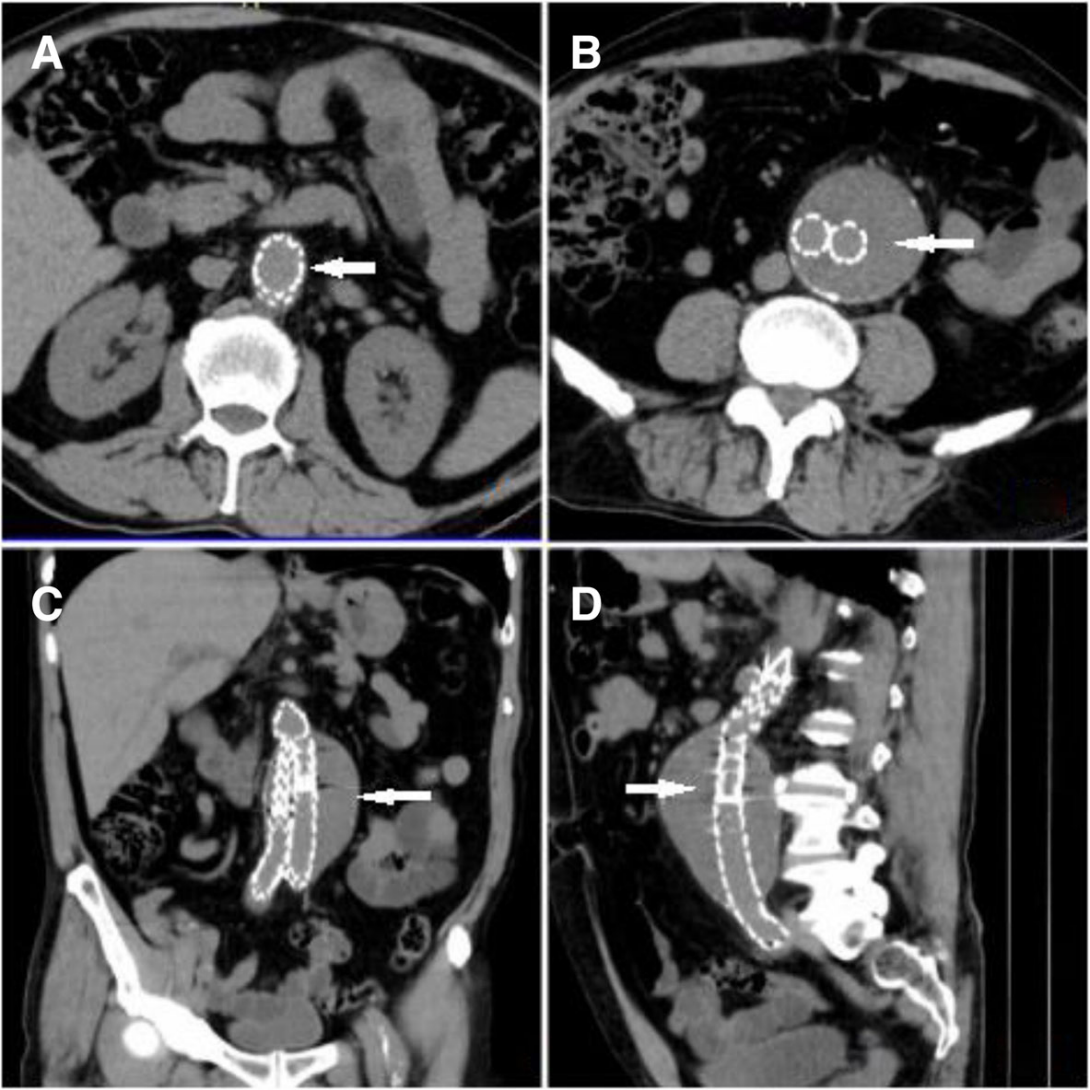
**a** Abdominal computed tomography transverse section after endovascular surgery. Endovascular prosthesis implantation is observed. **b** Aneurysmal stent implantation is observed. **c** Coronal section with implantation of prosthesis. **d** Sagittal section with implant of prosthesis. The prosthesis is marked with *white arrows*

The patient was a 69-year-old Hispanic man with no family history of cardiovascular or hematological diseases. He was a heavy smoker with a history of 15 pack-years. He was retired and not an alcoholic. He had a history of arterial hypertension, revascularized ischemic heart disease, and aortic valve replacement 10 years earlier, in addition to five coronary stents, chronic peripheral arterial disease of the lower limbs, and an aneurysm of the infrarenal aorta and left primitive iliac artery (Fig. 1a, b). He underwent endovascular surgery with an Endurant II stent (Medtronic, Minneapolis, MN, USA) and a successful right hypogastric artery embolization (Fig. 2a–d). There were no complications in the postoperative period; he received ambulatory anticoagulant therapy with low-molecular-weight heparin and subsequent change to warfarin, but with little adherence to the initial treatment. In addition, he received atorvastatin 40 mg daily, acetaminophen 1 g every 8 hours, clonidine 150 mg every 8 hours, losartan 50 mg every 12 hours, nimodipine 60 mg every 4 hours, phenytoin 300 mg/night, and bisacodyl 10 mg daily. He did not receive any antibiotics before the surgery; after the surgery, he received norfloxacin 400 mg every 12 hours for 7 days to treat a urinary tract infection.

**Fig. 3:**
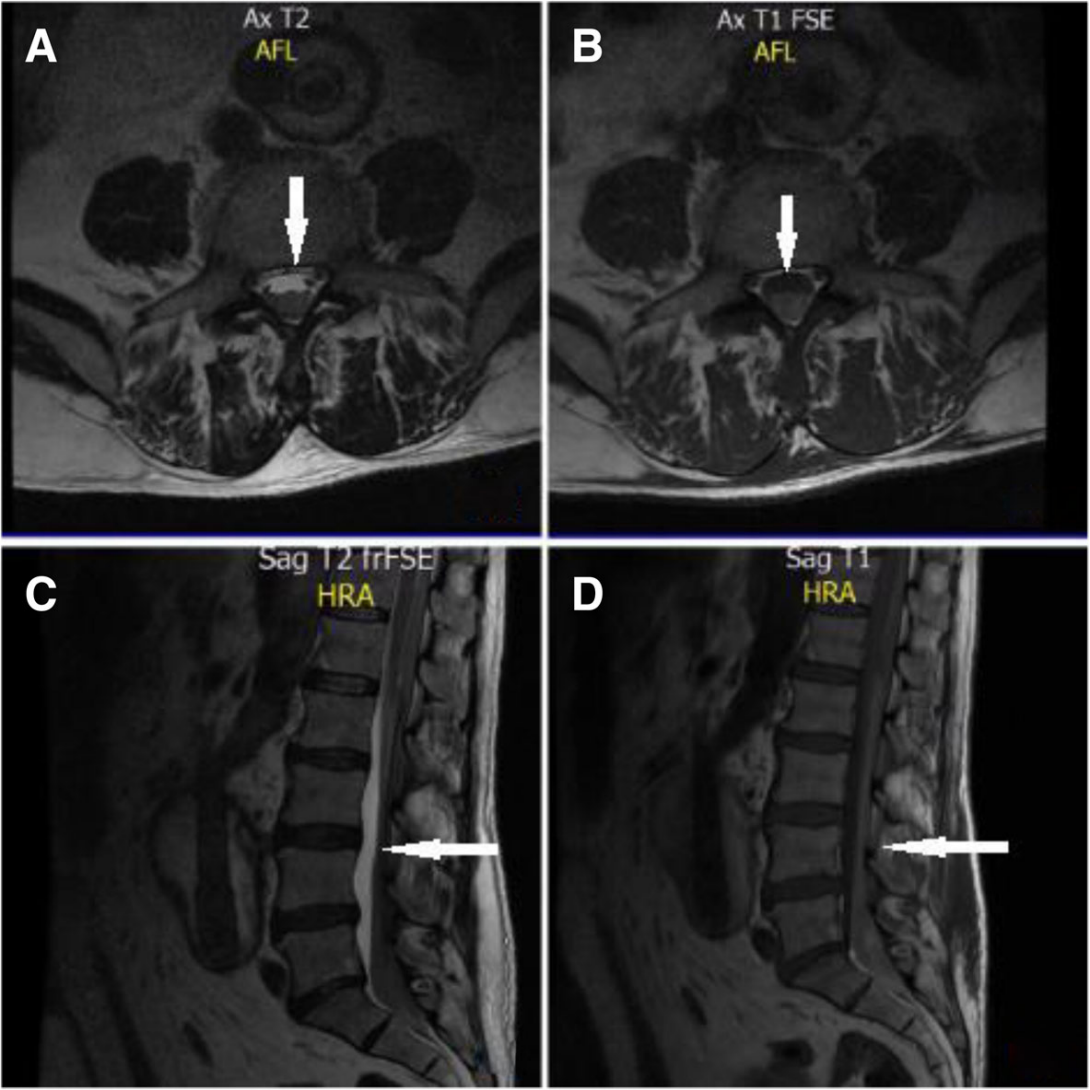
**a** Magnetic resonance imaging (MRI) T2-weighted transverse section. A hyperintensity zone is observed showing spinal infarction at the T10 level. **b** MRI T1-weighted hypointensity showing spinal cord infarction. **c** MRI T2-weighted sagittal section showing extension of the medullary infarction with hyperintensity zone. **d** MRI T1-weighted image showing extension of the medullary infarction with hypointensity zone. The area of infarction is indicated by the *white arrow*

Two months after surgery, he was admitted to the emergency room with blood pressure of 97/52 mmHg, heart rate 79 beats/minute, respiratory rate 20 breaths/minute, and temperature of 36 °C. He presented with sudden pain in his lower left limb with signs of ischemia (absence of popliteal and pedis pulses, paleness and coldness of the extremity, motor and sensitivity loss), a partially normal neurological examination regarding orientation, with compromise of superficial and deep sensitivity of the lower limbs. He had laboratory test results of white blood cell count of 15,2 x10^3/uL, neutrophils 82%, lymphocytes 17%, hemoglobin 15.2 g/dL, hematocrit 44.5%, platelets 154 x10^3/uL, blood urea nitrogen 14.3 mg/dL, creatinine 0.87 mg/dL, and initial international normalized ratio (INR) in subtherapeutic range (INR 1.05–1.95). He was diagnosed with an exacerbation of his chronic peripheral arterial disease with an arterial duplex of his lower limbs, showing moderate atheromatous process of lower limb arteries, with acute left femoral popliteal artery occlusion from its origin extending to the anterior and posterior tibial arteries and pedis artery. The patient was initiated on intravenous unfractionated heparin (UFH) and dual antiplatelet therapy achieving anticoagulation goals with subsequent gradual improvement of limb ischemia. After 48 hours of observation, he had a sudden pain in his lumbar region associated with absence of sphincter control and loss of strength of his lower limbs with a Medical Research Council scale score of 0/5 (complete paralysis). Contrast-enhanced magnetic resonance imaging (MRI) was performed, which revealed extensive dorsal myelopathy from T3–T4 to T11–T12 (Fig. 3a–d) of compressive and/or ischemic nature, and extensive spinal cord infarction was determined. Twenty-four hours later, despite the established antihypertensive treatment, he presented with a hypertensive emergency with acute target organ damage. Anti-ischemic management was initiated, and it was decided to stop heparin due to possible excessive anticoagulation. During the evolution of this presentation, he had headaches; hence, cerebral computed tomographic angiography was performed, which showed supra- and infratentorial intraparenchymal hemorrhage and thalamic hematoma. An intensive care unit stay as well as rehabilitation for the subsequent management of his symptoms and stabilization of comorbidities was required.

After commonly agreeing with the family, he was discharged for home healthcare planning. When discharged, the patient’s pure motor deficit in his lower extremities persisted, along with absence of sphincter control and presence of mild cognitive impairment, specifically in memory. Seven months after outpatient treatment, with progressive worsening of complications of his disease with depression, recurrent infections, and skin ulcers, the patient died.

## Discussion

A 69-year-old man with multiple comorbidities had an infrarenal aortic aneurysm treated with endovascular surgery and hypogastric artery embolization without complications. He received anticoagulant therapy with little adherence to the treatment. Two months after surgery, he was admitted to the emergency room with an exacerbation of his chronic peripheral arterial disease. Treatment with intravenous UFH and dual antiplatelet therapy achieved anticoagulation goals. However, 48 hours later, he was diagnosed with an extensive dorsal myelopathy of compressive and ischemic nature, as well as an extensive spinal cord infarction, despite the management with dual anticoagulation and antiplatelet therapy, making this case report an important contribution to the medical literature. There is no specific data for patients who undergo this type of endovascular correction; therefore, it is necessary to continue with clinical investigations that provide more clarity about the onset of anticoagulation, antiplatelet management of dual schemes with the purpose of decreasing the risk of complications.

The endovascular treatment for an aortic aneurysm, both thoracic and abdominal, has increased due to the multiple benefits it offers, being a minimally invasive technique that does not require general anesthesia. The costs associated with the intervention are minor, and the intervention can be performed by different specialties such as endovascular surgery or interventional radiology [1]. However, some reports indicate concern with the high probability of generating embolization at any level secondary to the endovascular approach compared with open surgery [5].

Medullary ischemia after a surgical intervention in the infrarenal aorta, according to some cohorts, has an extremely low reported incidence, up to 0.21% [3]; however, in other reports, it has reached 13.8%, increasing if the patient presents with risk factors such as hypotension during surgery [6, 7], history of repair with a previous endoprosthesis, size of the aneurysm, and surgical technique [8]; using minimally invasive strategies such as the use of catheters and other artifacts can have prothrombotic effects [5]. It is believed that neurological damage after an aortic intervention is secondary to hypoxic damage of the spinal cord and the generation of free radicals in addition to reperfusion edema [9].

The clinical presentation of this complication is unspecific. It has been described that patients arrive with symptoms that suggest abdominal disease because many cases start with abdominal pain associated with nausea, vomiting, and low back pain, which confuses the clinical presentation with a wide range of diagnostic possibilities of abdominal origin [10, 11]. Subsequently, the neurological compromise is generated; however, the onset of this compromise is variable [12]. In one case, it was reported that complete paraplegia was established within the first 18 hours of admission to the emergency room [13], and other clinical cases report that the appearance of this complication was later, between 2 and 10 months after the intervention [14, 15].

The diagnosis of this complication is confirmed by imaging, with MRI being the best tool available with a very high specificity [2]; however, not all cases present a visible alteration on the MRI scan, and it should be suspected on the basis of the patient’s clinical presentation [15]. The diagnosis of spinal cord ischemia in a patient undergoing abdominal aneurysm repair with stent placement is an interesting clinical challenge. In our patient’s case, he was not in therapeutic anticoagulation ranges at admission, and despite initiating anticoagulant and antiplatelet treatment obtaining early therapeutic goal values, he finally presented with the ischemic event at the spinal level associated with a hemorrhagic cerebrovascular event.

## Conclusion

Several ideal conditions to minimize the risk of complications in patients undergoing this type of procedure have been proposed in the literature; nevertheless, there is no clarity on the need or indications for the use of anticoagulant or antiplatelet therapies with good clinical evidence in this type of patient. However, it is considered that patients should receive treatment with at least one antiplatelet medication after surgery to reduce potential risks associated with it. In this regard, the most frequently used antiplatelet agents, aspirin and clopidogrel, have been discussed because both have shown considerable effects in the reduction of ischemic events [4]. However, there is no specific data for patients who have undergone this type of endovascular correction; therefore, it is necessary to continue with clinical investigations that give more clarity about the onset of anticoagulation and antiplatelet management in dual schemes with the purpose of decreasing the risk of complications.

## Data Availability

Data sharing is not applicable to this article, because no datasets were generated or analyzed during the current study.
